# Climate Change, Health and Existential Risks to Civilization: A Comprehensive Review (1989–2013)

**DOI:** 10.3390/ijerph15102266

**Published:** 2018-10-16

**Authors:** Colin D. Butler

**Affiliations:** National Centre for Epidemiology and Population Health, Australian National University, Canberra 0200, Australia; colin.butler@anu.edu.au; Tel.: +61-428-811-675

**Keywords:** citation analysis, civilization collapse, climate change, comprehensive review, conflict, existential risk, famine, global warming, global health, migration

## Abstract

*Background:* Anthropogenic global warming, interacting with social and other environmental determinants, constitutes a profound health risk. This paper reports a comprehensive literature review for 1989–2013 (inclusive), the first 25 years in which this topic appeared in scientific journals. It explores the extent to which articles have identified potentially catastrophic, civilization-endangering health risks associated with climate change. *Methods:* PubMed and Google Scholar were primarily used to identify articles which were then ranked on a three-point scale. Each score reflected the extent to which papers discussed global systemic risk. Citations were also analyzed. *Results*: Of 2143 analyzed papers 1546 (72%) were scored as one. Their citations (165,133) were 82% of the total. The proportion of annual papers scored as three was initially high, as were their citations but declined to almost zero by 1996, before rising slightly from 2006. *Conclusions*: The enormous expansion of the literature appropriately reflects increased understanding of the importance of climate change to global health. However, recognition of the most severe, existential, health risks from climate change was generally low. Most papers instead focused on infectious diseases, direct heat effects and other disciplinary-bounded phenomena and consequences, even though scientific advances have long called for more inter-disciplinary collaboration.

## 1. Introduction

In 1988 the leading climate scientist James Hansen, of the National Aeronautics and Space Administration, with three other senior researchers, testified to a U.S. Congressional committee that it was 99 percent certain that the warming trend in Earth’s temperature that was then observed was not natural variation but was caused by the accumulation of carbon dioxide and other “greenhouse” gases. This testimony was reported prominently in the New York Times [1,2]. Hansen was criticized then, and many times since, for his “adventurous” interpretation of climate data, however the publicity which followed his testimony, itself reflecting a decade of growing agitation about the geo-political impacts of climate change [2] may have influenced health workers to think more deeply about the issues. In any case, within a year, a Lancet editorial discussed health and the “greenhouse effect” [3], possibly the first such publication in a health journal, eight years after a chapter concerning climate change and parasitic disease appeared [4]. At least six other chapters on this topic were published in the 1980s, as well as at least two reports. For details, see [5]. Two other journal articles concerning climate change and health were also published in 1989 [6,7].

The 1989 editorial stated “global warming, increased ultraviolet flux, and higher levels of tropospheric ozone will reduce crop production, with potentially devastating effects on world food supplies. Malnutrition (sic) might then become commonplace, even among developed nations, and armed conflicts would be more likely as countries compete for a dwindling supply of natural resources” [3]. In the New England Journal of Medicine, Leaf warned, also in 1989, of sea level rise, especially in the south-eastern U.S. state of Florida, higher precipitation, millions of environmental refugees, an increased risk of drought and the possibility that warming at higher latitudes would not fully compensate any climate change related loss of agricultural productivity towards the equator [6]. The third paper published that year [7] was even more direct, warning of “catastrophic” consequences to human health and well-being.

In the early 1990s, warnings of potentially catastrophic consequences of climate change continued to dominate. Yet, by the turn of the millennium, the author had formed the impression that the scientific publishing milieu was becoming less receptive to the message that climate change and other forms of “planetary overload” [8] pose existential, civilization-wide risks. This was disturbing, as my own confirmation bias seemed to support the case that the evidence of existential risk was continuing to rise [9,10].

That the health risks from climate change are indeed extraordinarily high was stressed in the 2009 publication of the lengthy (41 page) article by the Lancet and University College London Institute for Global Health Commission, which described climate change as the “biggest global health threat of the 21st century” [11]. Yet, although this paper attracted considerable attention at the time, the long-term outlook for climate change and health has since continued to deteriorate.

By existential, I mean related to the word “existence”. But it is not the continued existence of Earth that is in doubt, but instead the existence of a high level of function of civilization, one in which prospects of “health for many” (though no longer “health for all”) are realistic and even improving [12]. Existential risk does not necessarily mean that global civilization will collapse. Nor does it exclude pockets of order and even prosperity enduring for generations, from which global or quasi-global civilization may one day emerge, provided worst case scenarios are avoided, such as runaway climate change and nuclear war leading to nuclear winter [13]. Compared to today, such prospects should be recognized as catastrophic. Unchecked climate change could generate similar, or bleaker, global futures. Seeking to minimize such possibilities should be seen as a major responsibility for all workers concerned with sustaining and improving global public health.

There is reticence [14,15], shared by many authors, reviewers, journals, funders and media outlets to discuss the possibility of such existential risks. Nonetheless, the consequences for health are so vast that discussion is warranted. This paper seeks to do that, in the process conducting the largest review on the topic of climate change and health yet to be published.

### 1.1. Climate Change Science, Risk and the 2015 Paris Agreement

The scientific knowledge that gases, accumulating mainly from the burning of fossil fuels and the clearing of forests, add to the natural “greenhouse effect” has been known since the 19th century [16]. In 1957 scientists observed “human beings are now carrying out a largescale geophysical experiment of a kind which could not have happened in the past nor be reproduced in the future. Within a few hundred years we are returning to the air and oceans the concentrated organic carbon stored over hundreds of millions of years” [17].

In 2015 the Paris climate change agreement, negotiated by representatives of 196 parties (195 nations and the European Union) committed countries (thus, effectively, civilization), upon ratification, to actions that would seek to restrict average global warming to “well below” 2 °C above “pre-industrial” levels and to “pursue efforts” to limit the rise to 1.5 °C. The text of the Paris Agreement defines neither the pre-industrial temperature nor the time for this baseline, but most experts agree that it means the temperature in the late 18th or 19th century, soon after the start of the industrial revolution, when coal burning increased. This time is after the end of the Little Ice Age, which itself was accompanied by a rebound in average temperatures, independent of the slow rise in greenhouse gases (chiefly methane and nitrous oxide as well as carbon dioxide) that occurred throughout the 19th century. Estimates of global warming for the period 1861–1880 until 2015 range from 0.93 °C [18] to 1.12 °C [19].

Although the goal of 1.5 °C is widely known, there is less understanding that meeting this challenge would not guarantee safety from a climate change perspective [20]. Indeed, if it were to be more widely accepted that climate change has already contributed to the Syrian war [21,22], to the rise in global food prices which accompanied the 2010 drought and heatwave in Russia [23,24], and the 2018 wildfire season in the Northern Hemisphere, then the threshold of danger might already be widely seen as having long been exceeded.

In recent years the science concerning the physical impacts of climate has continued to expand and to disturb. Average global temperatures continue to rise [25], apparently in a process more “stepped” than as a trend [26] with record average global heat in both El Niño and La Niña years. Loss of ice from both Antarctica and Greenland is increasing and the rate of sea level rise is consequently accelerating [27]. Property values in parts of the U.S. East Coast may soon fall, due to sea level rise [28]. There is growing concern about more intense rainfall [29,30], fires worsened by heat and drought [31], a weakening Gulf Stream [32] and increased sinuosity of the jet stream, which can cause unusual cold at lower latitudes, even if the average global temperature is rising [33,34]. The projected trend toward a weaker and poleward-shifted jet stream is also consistent with projections of a significantly increased risk of worsening extreme heat and dryness in the Northern Hemisphere [35].

There is also growing evidence of greenhouse effect-intensifying feedbacks in the Earth system [36] that might release enormous quantities of carbon dioxide and methane, independent of fossil fuel combustion, agriculture or deforestation, from sources including warming tundra and increased fires, both of peat and forests [37,38]. Such releases could dwarf the climate saving made possible by the putative implementation of the Paris climate agreement. The strength of the oceanic carbon sink is also weakening [39]. If this intensifies it is likely to accelerate warming of the atmosphere, ocean and land.

### 1.2. Interaction, Attribution, and Causation

All, or virtually all, environmental health effects interact with social and technological factors as well as other “purely” environmental determinants. For example, the effects of heat upon individual health are influenced by temperature, humidity, exercise, hydration, age, pre-existing health status, and also by occupation, clothing, behavior, autonomy, vulnerability, and sense of obligation. Does the person affected by heat, perhaps a brick maker in India, have the capacity to regulate her heat exposure; or might they be an elite athlete or emergency worker voluntarily pushing their limits? Other factors influencing the heath impact of heat include housing quality, the presence of absence of affordable air conditioning and energy subsidies, if any. In turn, these factors are influenced by governance and socio-economic status. Thus, the health-harming effects of heat can be seen to have many contributing causes, of which climate change is only one. As McMichael (and before him David Hume, among others) pointed out, causal attribution is to an extent philosophical; it is influenced by the “focal depth” of the examiner’s “causal lens” [40]. Consider a mass shooting in a school: Some will see underlying social and legal factors as contributing; others may see only the shooter. Yet, a major role and goal of public health is to seek to identify and reduce “deep” or “underlying” causes [41]. A world in which only the most “proximal” causes are identified will not function well.

Attributing the fraction of human-caused (anthropogenic) climate change to physical events such as storms, floods and heatwaves is similarly contested and assumption-dependent. The contribution of climate change to more indirect, strongly socially mediated effects such as migration, famine or conflict is even more difficult and contentious [22,42,43]. Perhaps in part because of these causal complications, issues such as famine, genocide, large-scale population dislocation and conflict have, with rare exceptions [44], been peripheral to public health. This is despite the obvious large-scale adverse health effects of these phenomena.

Rigorous methods have been developed to detect and attribute the health effects of phenomena that are more directly or obviously related to climate change, such as heat and infectious diseases [45]. However, excessive caution risks a type II error, the overlooking of genuine effects [46,47]. To reduce this risk, the authors of a recent study on attribution acknowledged the role for “well-informed judgments, based on understanding of underlying processes and matching of patterns of health, climate, and other determinants of human well-being” [45]. This paper makes many such judgments.

### 1.3. Integrative Risk and the Sustainability of Civilization

Publications in health journals about nuclear war and health date at least to 1962 [48]. In 1992 the Union of Concerned Scientists coordinated the “World Scientist’s warning to humanity”, signed by over 1700 leading scientists (but no public health workers) [49]. This warning was repeated in 2017, with far more signatories (including many health workers) [50].

Many authors outside health have warned of the fragility of modern civilization [51,52]. However, comparatively few writers with a health background have contributed [9,10,53,54]. Tony McMichael, who led the first Intergovernmental Panel on Climate Change chapter on health [55] frequently wrote and spoke of eroding “life support mechanisms” [56,57], a term probably introduced into the health literature in 1972 by Sargent [58]. Certainly, McMichael wanted to convey, when using this term, a profound risk to human well-being and health.

If civilization is to collapse then effects such as conflict, population displacement and famine are likely to be involved. A heatwave, on its own, is unlikely to cause the collapse of civilization, nor even ruin an economy for a decade. It needs social co-factors to do this. For example, a series of heatwaves damaging crop yields and contributing to internal migration has been postulated as contributing to the Syrian civil war that started in 2011 [21,22,59,60,61,62]. Prolonged heat, especially if in a humid setting, could cause some regions to be completely abandoned [63,64,65]. 

A severely damaged health system, allied with worsening undernutrition and poverty, could provide a milieu for a devastating epidemic, including a resurgence of HIV/AIDS [66]. An increase in infectious diseases, if of sufficient scale, could contribute to integrative cascades of failure generating regional or even global civilization collapse. Infectious diseases, as well as unfavorable eco-climatic change, contributed to the collapse of the Roman Empire [67].

While such consequences may seem far-fetched to some, the prospect of sea level rise of one meter or more by 2100 (perhaps sooner), proliferating nuclear weapons, millions of refugees, xenophobia and tribalism which limits integration, and growing cases of state failure is disquieting. Few, if any, formal scenarios, as exercises by senior scientists, are as bleak, but funding and other pressures constrain the realism of such exercises [15]. Already, the number of forcibly displaced people exceeds 68 million [68], a rise that has been linked with tightening limits to growth, including climate change [69].

It is stressed, again, that the idea that any single climate related event, such as heat, drought, sea level rise, conflict or migration will cause the collapse of civilization is simplistic. It is far more plausible to conceive that collapse (or quasi-collapse) could arise via a “milieu” of multi-factorial risk, enhancing, inflaming and interacting with climate change and other factors [43,70].

### 1.4. Hypothesis

This article seeks to test the hypothesis that the early literature relevant to climate change and health was more willing to describe catastrophic, potentially civilization disrupting health effects including famine, mass migration and conflict than it was to become, at least until 2014.

## 2. Methods

To explore this hypothesis, a database of articles relevant to climate change and health was assembled, relying mainly on PubMed and Google Scholar. This had six steps (see Appendix for details). Due to limited resources, the main search was restricted to the period 1980–2013, and the terms “climate change” and health or “global warming” and health. After eliminating duplicates, remaining papers were checked to see if they met eligibility criteria (see Box 1).

Box 1 **inclusion and exclusion criteria.**

**Included:** Articles, editorials, commentaries, journalistic pieces with bylines.

**Excluded:** Reports, books, book sections including e-chapters, letters, factsheets, monographs, un-credited journalistic entries, non-English publications, papers concerning stratospheric ozone depletion, podcast transcripts, journalistic pieces that could not easily be recovered.

The search was not restricted to health or to multidisciplinary journals. However, papers outside health journals had to meet more exacting requirements to be included. They had to include health (or a synonym such as nutrition) in their title, abstract, keywords or text, even if they focused on an effect with health implications, such as population displacement, conflict or food insecurity.

The title of each identified paper was read, followed by the abstract of each paper, assessed as possibly eligible. If a score was still unclear, the full text was obtained and searched for words and phrases that suggested a broader interpretation of the indirect effects of climate change, such as “population displacement”, “migration”, “conflict”, “war”, “famine”, and “food insecurity”.

Eligible papers were scored as one if they exclusively concerned an effect other than conflict, migration, population displacement or large-scale undernutrition or famine. They also needed to exclude statements (even if introductory) such as “climate change has been recognized as the greatest risk to health in the 21st century”.

Papers were scored as two if they either mentioned such an effect and/or contained statements recognizing the potentially enormous scale of the health impacts from climate change. A synonym for this understanding was the phrase eroding “life support mechanisms”.

Papers were scored as three if they included a more detailed explanation or assertion of the future (or current) existence and importance of conflict, migration or famine, perhaps suggesting an interaction among them. A score of three was more likely if they also warned of the general severity of climate change. The score was also influenced by the tone of the language, and the space devoted to these issues (see Appendix for further details).

In addition, PubMed was searched for papers published from 2014–2017 matching the criteria “climate change” and “health”. A sample of 156 of these articles was randomly selected, approximately 5% in each year, after the elimination of a proportion of ineligible articles. Each was then scored, using the method described above for papers published from 1989 to 2013 (inclusive). Bootstrapping was then used to estimate the average score and 95% confidence interval of these articles, by taking ten thousand resamples, each of 156 papers, with replacement from this set (so that in each iteration some papers will appear more than once, while others will not appear at all). 

## 3. Results

A total of 2143 unique articles and journalistic essays satisfied the inclusion criteria, for the period 1989–2013 inclusive. The full database is available in the supplementary material. This shows the year, lead author (at least), journal, title and primary search method. It also lists the number of Google Scholar citations and the date these were identified. Table A1 (Appendix) tabulates the primary search method of papers, by each year. 

**Figure 1:**
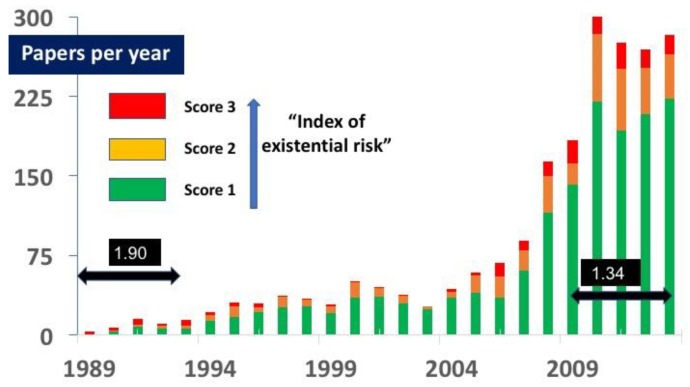
Number of papers in each category. Since 1989 the number of papers concerning climate change and health has expanded considerably, particularly since 2008. As this article did not review the entire literature, the actual number of papers published, even in English, is more than shown. The average score of these papers declined from 1.9 in the first quintile to 1.34 in the final five years.

No paper published before 1989 was eligible for retention in the final database. One potential publication [71] was cited by Kalkstein and Smoyer [5] as published in 1988, but it could not be located. About half the total papers (1142 or 53%) were published since 2009 (see Figure 1). Most papers (1546 papers, 72%) were scored as one, while only 189 (3.3%) were scored as three. The difference in these scores is statistically significant (*p* < 0.01 ANOVA). The average score of these 2143 papers was 1.37 (see Table A2 in Appendix).

The increase in the size of literature reflects growing awareness of the risks to health from climate change. Over 50% of the papers published in the first quintile (1989–1993) were scored as two or three, although the total number in that time (27) was small (see Figure 1). Since 1993 the majority of papers have focused on effects such as heat, infectious diseases, allergies or asthma. The number of papers scored as two or three increased slightly after its trough (23%) in the third quintile (1999–2004) but was only 26% for 2009–2013 inclusive.

**Figure 2:**
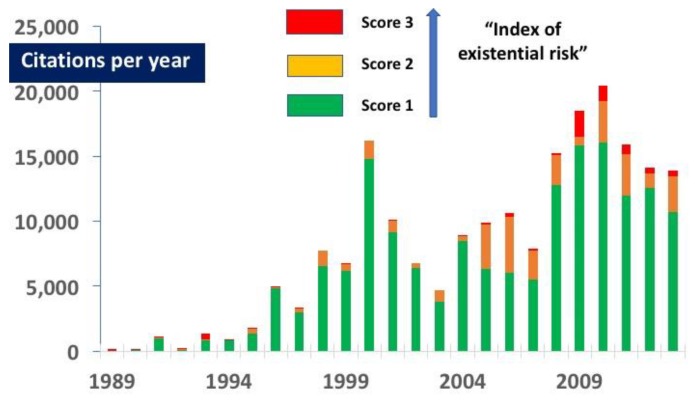
Number of citations per annum for each score of paper. Most citations were for papers scored as one. Note that in 2005–2007 three extensively cited papers were scored as two (these are discussed in the Appendix A).

Papers scored as three were particularly uncommon in the third quintile (1999–2003), representing only 2.6% of the total published papers in that period. Even in the first quintile (1989–1993) most citations were for papers scored as one (see Figure 2).

### 3.1. Citations

**Figure 3:**
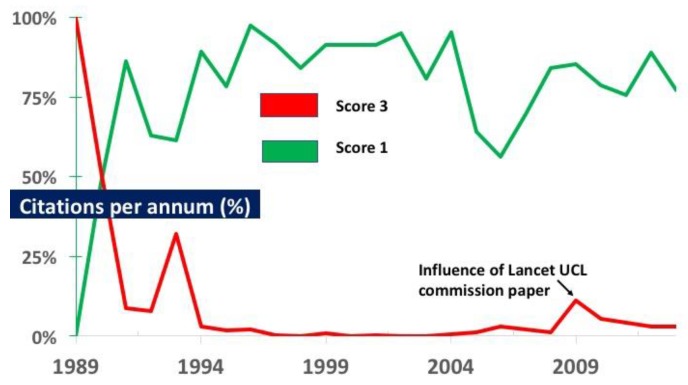
The proportion of citations each year for papers scored as one and three. Since 1991 most citations have been for papers scored as 1. The Lancet UCL paper published in 2009 [11] led to a resurgence of citations for papers scored as 3, but this effect declined. Three individual papers, each scored as two (published in 2005, 2006 and 2007), were disproportionately cited. In each year at least some papers scored two or three, but their proportion of citations fell steeply after the first quintile. In 2003 no paper was scored as three, and for almost a decade (1997–2005 inclusive) virtually no papers scored as three were cited.

Citation data were available for 2105 papers (98%). Over 201,000 citations were identified by Google Scholar (see Table A3 in Appendix). Thirty two percent of these citations were for papers published since 2009 (see Figure 2). Of these citations, the great majority (82%) were for papers scored as one, each of which was cited an average of 107 times. Papers scored 2 were cited an average of 73 times, representing 15% of the total. Papers scored as three were cited 35 times each on average and accounted for 3% of the total. The difference in these citation scores is also statistically significant (*p* < 0.01 ANOVA). Citations for papers scored as three from 1995 to 2008 inclusive were even lower, accounting for less than 1% of the total citations in each year of this period (see Figure 3). The fraction of the literature discussing existential risk remained lower in the last 5 years of this database than in the first five years (see Figure 1). The shift in the ratio of annual citations from the early period to the more recent years is evident in Figure 3. Until 1991, the majority of citations were for papers scored as three. From 1994 the fraction of citations for papers scored as three was almost zero (3% or less) in every year until 2009. In 2013 it again fell to 3%. 

### 3.2. Coverage of Topics

All papers published in 1989 discussed multiple potential health effects of climate change. However, from 1990, journal articles focusing exclusively on infectious diseases and climate change appeared [72,73,74]. Early papers also focused on heat [75] and allergies [76]. From 2000, the foci of concerns expanded greatly. Additional topics included reduced micronutrient concentrations in food [77], asthma [78], thunderstorm asthma [79], chronic diseases and obesity [80], toxin exposure (such as from increased concentrations in Arctic mammals [81] and increased algal blooms [82]), forest fires [83], mental health [84] and respiratory [85], cardio-vascular [86], renal [87], fetal [88], genito-urinal [89] and skin conditions [90]. By 2000, papers were also appearing arguing that the impact of climate change for malaria was overstated [91,92].

Articles also appeared on the impact of climate change on groups such as indigenous people [93], children [94], the elderly [95] and regions and locations, including cities [96], the Arctic [97] and small island states [98] as well as many individual nations. Other themes appeared, including on how the health sector might reduce its carbon footprint [99], on “co-benefits” [100], on climate change as a great opportunity to improve public health [101], on medical education [102], pharmaceuticals [103] and on the health risks of adaptation and geoengineering, including of carbon capture and storage [104].

### 3.3. The Leadership Role of Some Journals

Many journals played prominent, even campaigning roles, especially the Lancet, BMJ and Environmental Health Perspectives. Several journals had special issues, including Global Health Action, the American Journal of Preventive Medicine, the Asia Pacific Journal of Public Health and Health Promotion International. Seven journals published at least 28 articles each, including editorials and news items (see Table A4 in Appendix). At least 34 journals published editorials, which, with an average score of 2.2, were more likely to be scored as two or three than journal articles (average score 1.3). News items and other journalistic pieces had an average score of 1.6. At least 21 articles were published in nursing journals, with an average score of 1.67.

### 3.4. Papers for the Period 2014–2017

A total of 3377 papers were identified by PubMed as published from 2014–2017. Of these, 346 were found to be ineligible, although the true number would be higher, if all candidates were examined. Of the potentially eligible remainder, 113 papers were published in 2018, but recorded by PubMed as e-published in 2017. Slightly over five percent of the articles for each year was randomly selected, resulting in 156 articles (see Table A5 in Appendix). Their average score and 95% confidence interval, estimated by bootstrapping, was 1.29 (95% CI 1.21–1.39) (see Figure A2 in Appendix). Details of these 156 papers are in the supplementary material. Note that their citations were not checked.

## 4. Discussion

This paper describes the first published analysis of the extent to which the literature on climate change and health has described or in other ways engaged with “existential” risk. By including 2000 articles, 60 editorials and 83 news items (2143 “papers” in total) on climate change and health, it is by far the largest review of the climate change and health literature to have so far been published. Lack of resources currently prevents an extension of the fuller analysis to more recent years. However, a randomly selected sample of 156 articles for papers identified by PubMed as published in the period 2014–2017 found that these papers had an average score lower than the average score for any quintile from 1989–2013, other than for 1999–2003 (see Table A2 and Table A5 in Appendix).

Several systematic and other reviews of topics related to climate change and health have been published, but on a much smaller scale, and with different research questions. Ford and Pearce systematically reviewed 420 papers, published between 1990 and 2009, exploring the topic of climate change vulnerability in the Canadian western Arctic [105]. Two systematic reviews concerned heat. Huang et al. [106] searched for papers published between 1980 and July 2010, projecting the heat related mortality under climate change scenarios. Only 14 papers were included in their final analysis. Xu et al. [107] explored the relationship between heat waves and children’s health, but selected twelve, an even small number. A systematic review into dengue fever and climate change (for the period 1991–2012) included 16 studies [108].

Nichols et al. (2009) [109] undertook a systematic review on health, climate change and energy vulnerability, searching for papers published in English between 1998 and 2008. They retrieved 114 papers but included only 36 in their final analysis. Bouzid et al. (2013) undertook a “systematic review of systematic reviews” to explore the effectiveness of public health interventions to reduce the health impact of climate change [110]. This identified over 3100 unique records, but of these, only 85 full papers were assessed, with 33 included in the final review.

This may also be the first review paper concerning climate change and health to use a citation analysis [111] as an indicator of influence. Citations in Google Scholar were used for convenience and cost. Although such citations are prone to error, and include essays in the gray literature, they still reflect influence. Some reports in the gray literature may be more widely read and more influential than more scholarly work.

### 4.1. Selection and Other Forms of Bias

A systematic review was not undertaken. However, all papers identified by searching using PubMed and at least 100 papers for each year identified by Google Scholar were considered for inclusion. The search term relevant to health was restricted to a single word, rather than synonyms such as “disease”, “morbidity”, “illness”, or “mortality”. Undoubtedly, a search using additional terms will identify more papers, as would a systematic review.

To examine the possibility that a more extensive search strategy would alter the conclusions, PubMed was also searched for the terms “climate change” and “morbidity” for papers published in 2013. This strategy identified 261 papers, compared to 496 when searching for “climate change” and “health”. Of these 261 papers, 30 had not previously been identified by the other search methods used, and met the other inclusion criteria. However, all of these additional papers were scored as one. Their inclusion in the final analysis was considered likely to bias the paper away from the null hypothesis, by accentuating the fraction of papers not scored as two or three. This bias towards papers scored one (i.e., identified by searching for “morbidity”) seems plausible because the term morbidity may be more likely to be associated with specific diseases than the term “health”. These papers therefore were not added to the analysis.

**Figure A1:**
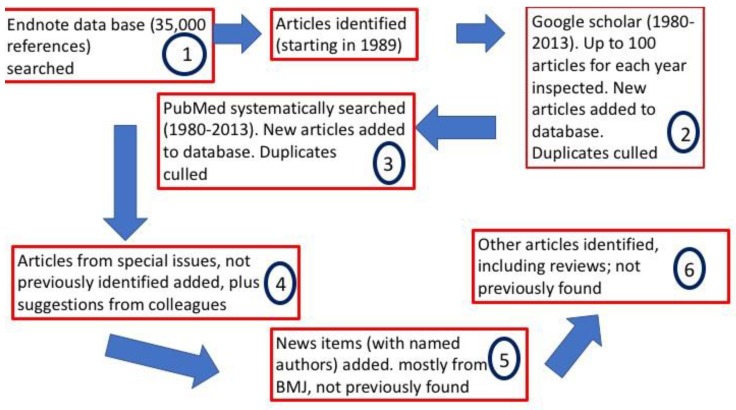
Outline of the six stage search strategy for papers published from 1989–2013.

**Figure A2:**
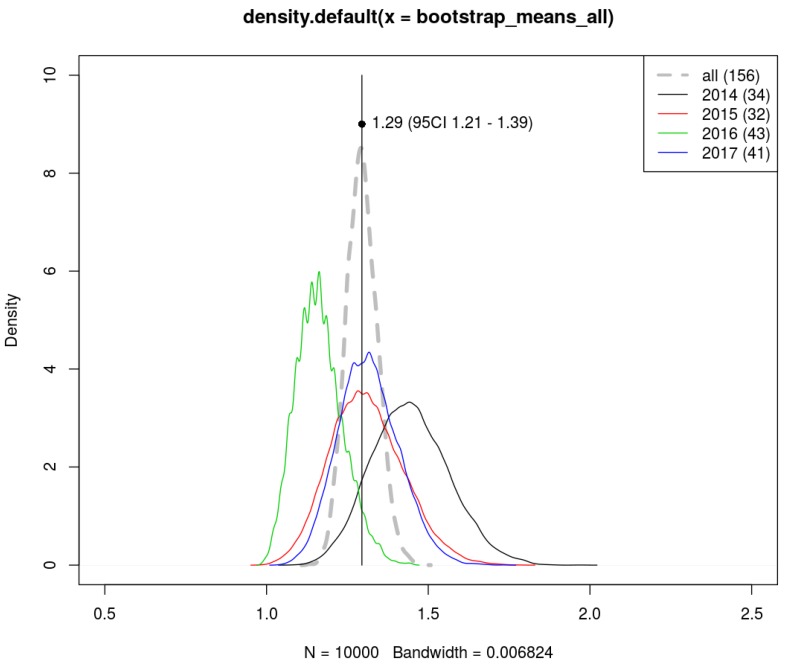
This shows the density of means and distributions for each year (2014–2017), based on 10,000 bootstrapped resamples (with replacement from the set for each year) and also for papers from 2013–2018 inclusive.

The search was supplemented by the addition of 17 papers first identified from the author’s own database, but not later found by the search strategy using Google Scholar or PubMed (steps 2–3) as described in Figure A1. Eight of these 17 papers, five of which the author wrote or co-wrote, were scored as three. Their average score was 2.17, far higher than for the balance (1.23). This group also includes two editorials, one published in the Lancet, one in the BMJ. The inclusion of one of these editorials (scored as three, published in 1989) has biased the findings in favor of the hypothesis that highly scored papers were more common in the early period of this literature. Note, however, that no citations were recorded for this editorial.

The inclusion of these higher scoring papers later in the period of analysis has biased the result to the null, that is, away from the hypothesis that fewer such papers were published from about 2000. The most influential of these 17 papers, judged by Google Scholar citations, was cited 272 times. It was the first to report that rising levels of carbon dioxide depress micronutrient concentrations in food [77]. The other 16 papers were cited 405 times between them, an average of 25, which is low compared to the average citation number (94). Twenty eight other papers were included, mostly identified from special issues. Their average score was 1.9. One paper was identified post-review, by chance. It was scored as two (perhaps generously) and was included because it was judged that to exclude it would bias the result away from the null hypothesis.

Bias is also likely to have been introduced in the scoring process, but not to the extent that it could challenge the main conclusions. The rigor of this paper would be improved if the scores could be checked by a third party, blind to the first score. Unfortunately, no resources were available for this purpose. Some classification errors are likely, especially for papers for which the author had no previous familiarity, and if published after 2009, when, due to time pressure, many papers were scored rapidly. On the other hand, in the process of ranking over 2000 papers the author became skilled at making rapid decisions, especially for most papers scored as one. The difference between papers scored one and two was generally more apparent than for papers scored between two and three. In cases of doubt a higher score was always selected.

The likelihood of bias and error is unlikely to explain the difference in the character of the papers in the early period and those which later dominated. Although the widely cited paper by Costello et al. [11] (1583 citations as of June 2018) may have refreshed appreciation of the potentially catastrophic nature of climate change, the majority of papers and their citations published between 2010 and 2013 continued to focus on specific issues. This trend appears to have persisted in the years since, judged by the analysis of a randomly selected sample, identified by PubMed as published between 2014 and 2018.

### 4.2. Reasons for the Apparent Conservatism of the Literature

There are several plausible, overlapping and interacting explanations for the decline in the proportion of papers scored as two or three (and for their comparatively fewer citations) following 1996, and also in the failure for papers published since 2009 to fully amplify the most severe warnings. One likely contributing explanation is self-censorship. The topic of climate change and health is unfamiliar territory for many health editors and writers. Climate change has become politicized in many English-speaking countries, especially in the U.S. and Australia. Although comparatively few health workers have expertise concerning climate change and health, the readership of some health journals seems judged, by their editor, to be skeptical of, or even to reject climate science. For example, one editor, defending the decision to publish a paper (scored, possibly generously, as two) [112] seemed almost apologetic, writing “On its face, the paper by Hess and colleagues is largely a political commentary and a departure from the types of articles found in Academic Emergency Medicine” [113].

Thus, for some health workers and editors, even broaching the topic of climate change and health may be a courageous act. The publication of papers in health journals that describe potential pathways that could threaten civilization would appear even bolder. It is unsurprising that such papers are still fairly uncommon, at least until 2014, and particularly in journals which do not yet have a long tradition of publishing papers or editorials on this topic.

In the early period of the climate and health literature (1989–1993) some of the most outspoken articles were editorials. Perhaps at that time, there was a certain sense of shock concerning climate change, which has since waned. It was also a time when concerns about overpopulation were slightly less taboo [114,115,116]. However, editorials in more years also tend to have a higher index of concern than other articles.

Another likely contributor to the comparative degree of restraint is the view, backed by some research, that an excess of fear is counter-productive [117]. However, the smell of smoke in a theater requires the sounding of a vigorous alarm. Compounding the difficulty of communicating the risk over climate change is the lag between the whiff of smoke and the onset of visible fire. Hansen warned of great danger over thirty years ago, and he, with others, have issued many warnings since [118]. Sceptics are still waiting to see the metaphorical “flames” of climate change, even disputing the link between literal flames (fires) and climate change. 

On the other hand, science, though not infallible, has delivered countless miracles such as antisepsis, anesthesia, penicillin and the jet engine. It has long warned of the physical changes of climate change. We who work in health should not be amazed if the predictions of climate and Earth scientists prove broadly accurate. Social science is less precise than climatology [43], however the links between food insecurity, drought, sea level rise, migration and, in some places, conflict are, also, surely not far-fetched. Papers that fail to express appreciation of the extraordinary risks we face as civilization may be judged by people of the future as having failed in their duty of care to protect health.

Another likely reason for the general restraint in the literature is the fragmentation of science and limited funding for multidisciplinary work. Comparatively few authors, other than if collaborating in large, multidisciplinary teams (rare for most authors primarily concerned with health), are rewarded or funded for thinking systemically. This problem is possibly worsening. Related to this, many recent papers are by sub-disciplines of health that have not previously published on the topic of climate change. Such papers are probably less likely to discuss existential risk.

As the effects of climate change have become increasingly clear the need for adaptation has become overwhelming. A stress on adaptation does not necessarily reflect any underestimation of the eventual severity of climate change. However, a stress on adaptation at the expense of mitigation may do so. In many countries, political leadership favors adaptation. 

## 5. Conclusions

In 1989, thirty two years after the International Geophysical Year, the first papers on global warming and health appeared in the world’s leading medical journals [3,6,7]. All three of these early papers warned of severe, even existential risk and were each scored as three.

In 1990 McCally and Cassel warned that “progression of these environmental changes could lead to unprecedented human suffering” [119]. Also, in 1990, Fiona Godlee, then deputy editor of the BMJ, wrote “Countries in the developing world would suffer both the direct effects of drought and flood and the knock-on effect of agricultural and economic decline in the West. The already present problems of feeding the world’s growing population would be compounded by the increasing numbers of displaced people unable to grow their own food” [120]. In 1992 Powles observed “It is possible that adverse lagged effects of current industrial (and military) activities will disrupt the habitat of future generations of our species through processes such as stratospheric ozone depletion, global warming and others as yet unpredicted” [121]. However, in the following years, this sense of urgency largely dissipated, until the long paper by Costello et al. in 2009 [11].

Conditioned by growing up during the Cold War, the author has long been apprehensive about civilization’s survival. However, my timeline for global health disaster has always been multi-decadal. Civilizational collapse, if it is to occur, will not necessarily be in my own lifetime [54]. My concerns are not based solely on climate change. Climate change, by itself, is most unlikely to cripple civilization. A well-functioning global society, motivated to do so, could easily eliminate hunger and poverty, not only today, but under all but worst-case climate change. Refugees from inundated islands, war-torn Syria or the drought-stricken Chad basin [122] could easily be accommodated in more fertile and more elevated parts of the world. Unfortunately, humans currently do not co-operate on such a scale, and this behavior may, in part, be driven by inborn, “hard-wired”, evolutionary-shaped traits [123]. If civilization is to endure we may need to collectively overcome our seemingly deep wiring for tribalism and separation.

## Supplementary Materials

The following are available online at http://www.mdpi.com/1660-4601/15/10/2266/s1.

## Appendix A

### Appendix A.1. Detailed Methods and Results

The search method had six steps (see Figure A1). Initial exploration used the author’s Endnote database, of over 35,000 references, to find relevant articles. The second step was to search, using Google Scholar, for up to the first 100 results for each year in the search period (1980–2013), using the terms “climate change” and health or “global warming” and “health”. For the first decade in which relevant articles were found (1989–1998) both pairs of terms were used, but from 1999 to 2013 inclusive, only the former terms were used (“climate change” and “health”). In the third step, the search was expanded by seeking the same terms, using PubMed, for the same period; 1980–2013 (inclusive). After eliminating duplicates, all remaining papers were checked to ensure that they met the eligibility criteria listed in Box 1. In stage 4, several papers were included if they appeared in special issues of journals, together with articles identified by PubMed, or suggested by colleagues. In stage 5, the BMJ database for news items about climate change and health was searched explored, because although PubMed found a few the proportion it identified was low. Finally, in stage 6, several other papers were found by chance, such as in reviews, in the references of cited papers, or by searching for other papers.

### Appendix A.2. Further Scoring Details

The following details are provided in order to provide additional information about the scoring process. It discusses the scoring process for three highly cited papers (from 2005–2007), each of which was scored as two. The first (cited 2059 times) had no mention of population displacement or conflict, but included the sentence “Projections of the effect of climate change on food crop yield production globally appear to be broadly neutral, but climate change will probably exacerbate regional food supply inequalities” [124]. This statement was assessed as accepting the possibility of a degree of food scarcity judged to be more severe than that described by many papers (particularly concerning the Arctic) which discuss a likely impairment in regional nutrition, but do not forecast insufficient calories or nutrients, let alone famine. Although the conclusion regarding overall global food security in this paper was reassuring, there are already four acknowledged famines in African nations and one in Yemen [125]. Any exacerbation of regional food supply inequalities is therefore likely to result in aggravated famines, unless future famines are eliminated; an unlikely prospect. Because this paper was cited so frequently a lower score would impact the overall result. If there is a bias from scoring this paper as two it is towards the null hypothesis.

In 2006 a widely cited paper [126] stated “Other important climatic risks to health, from changes in regional food yields, disruption of fisheries, loss of livelihoods, and population displacement (because of sea-level rise, water shortages, etc.) are less easy to study than these factors and their causal processes and effects are less easily quantified”. This is a more comprehensive list of civilization-endangering effects than the paper discussed above, but the language is restrained and brief. It was scored as a two.

In 2007 another widely cited paper included the sentences “Climate change will, itself, affect food yields around the world unevenly. Although some regions, mostly at mid-to-high latitude, could experience gains, many (e.g., in sub-Saharan Africa) are likely to be adversely affected, with impairment of both nutrition and incomes. Population displacement and conflict are also likely, because of various factors including food insecurity, desertification, sea-level rise, and increased extreme weather events” [127]. Of the three papers discussed here this provided the most comprehensive list of such effects and also explores their interaction. However, it did not speculate about civilization collapse, nor describe climate change as the biggest threat to global public health.

A gradient exists between papers scored two or three, rather than a clear threshold. Papers were not scored as three simply by including a more detailed explanation or assertion of the existence and importance of conflict, migration or famine, even if an interaction among them was suggested. They needed something extra. For example, one paper [128] stated (referring to Costello et al. [11]) “a watershed paper … suggests that climate change represents the biggest potential threat to human health in the twenty-first century … a recent report … also estimates that four billion people are vulnerable and 500 million people are at extreme risk”. This paper was scored as three even though the paper focused on medical education. Although the phrase “the biggest potential threat to human health in the twenty-first century” can, with repetition, lose its capacity to shock, its meaning, if taken literally, is surely sufficiently dire to be scored as three.

Another paper (scored as three) stated “global health, population growth, economic development, environmental degradation, and climate change are the main challenges we face in the 21st century” [129]. It also stated that “significant mass migration is likely to occur in response to climate change”.

The interpretation of papers was not excessively generous. For example, a paper that noted: “Changes in the frequency and intensity of extreme weather and climate events have had profound effects on both human society and the natural environment” was scored as one because there was no discussion of this aspect in the abstract or further in the text. It was also considered that the words “have had profound” was insufficiently clear. Nor did the paper discuss conflict, migration or famine.

In contrast, two papers about climate change and health in Nepal were scored as two, as they included the statements “Climate change is becoming huge threat to health especially for those from developing countries” (sic) [130] and “Climate change is a global issue in this century which has challenged the survival of living creatures affecting the life supporting systems of the earth: atmosphere, hydrosphere and lithosphere” [131].

### Appendix A.3. Sources (Detailed)

Seventeen articles were identified from the author’s database, but not found via PubMed or Google Scholar. Other sources are shown in Table A4.

**Table A1:** This shows the primary source of the 2146 included articles. 18 articles were from special issues, 5 were found accidentally, 1 was from a review and 1 was from a colleague. Many articles were found using multiple methods. The papers listed here in the GS column were not found by PM but may also have been identified by CB. Abbreviations: PM = PubMed, GS = Google Scholar, CB = Colin Butler.

Year	PM	GS	BMJ	Other	CB	Total
**1989**		2			1	3
**1990**	2	3		2		7
**1991**	14	1				15
**1992**	1	8			2	11
**1993**	7	7				14
**1994**	11	11				22
**1995**	13	17	1			31
**1996**	12	18				30
**1997**	16	21				37
**1998**	15	17	1		1	34
**1999**	10	19				29
**2000**	30	16	1	1	3	51
**2001**	34	8	2	1		45
**2002**	28	8	1		1	38
**2003**	17	8	1		1	27
**2004**	29	12	2			43
**2005**	35	18	4		2	59
**2006**	55	11	2			68
**2007**	67	16	3	3		89
**2008**	134	22	7	1	1	164
**2009**	109	54	18	1	2	184
**2010**	186	106	6	14	2	314
**2011**	176	93	5	1		275
**2012**	158	108	2		1	269
**2013**	154	126	2	2		284
**Total**	**1313**	**730**	**58**	**25**	**17**	**2143**

### Appendix A.4. Score, Citation and Journal Details

**Table A2:** This shows the number of articles and their average score for each quintile from 1989–1993.

Quintile	Number of Articles	Average Score
**1989–1993**	50	1.90
**1994–1998**	154	1.40
**1999–2003**	190	1.26
**2004–2008**	423	1.42
**2009–2013**	1326	1.34
**1989–2013**	**2143**	**1.37**

**Table A3:** This shows the number of papers and citations in each category divided into five quintiles for the 25 years of analysis. Note that in the third quintile (1999–2003) only 5 articles were ranked as three. Ironically, the paper scored as three in 2002 was a news item which quoted Andrew Sims, policy director of the New Economics Foundation as lamenting “Health is not even being talked about here [Delhi], although the potential health impact is a devastating one, almost unimaginable” [132].

	Papers Scored as 1		Papers Scored as 2		Papers Scored as 3	
	Number	Citations	Number	Citations	Number	Citations
**1989–1993**	23	1996	9	197	18	802
**1994–1998**	105	16,545	36	1910	13	172
**1999–2003**	146	40,352	39	3985	5	78
**2004–2008**	286	39,128	96	12,590	41	836
**2009–2013**	986	67,112	229	10,916	114	4748
**Total**	**1546**	**165,133**	**408**	**29,598**	**189**	**6636**

**Table A4:** Ten journals published at least 22 articles on climate change and health in the period 1989–2013.

Journal	Articles	Editorials	News Items	Total
**BMJ**	35	12	71	**123**
**Envtl Hlth Perspectives**	107		4	**121**
**Lancet**	55	8	2	**68**
**Int J Envtl Research & Pub Hlth**	53			**53**
**Global Health Action**	39			**39**
**Int J Biometeorology**	38			**38**
**PloS One**	28			**28**
**Int J Circumpolar Research**	26			**26**
**Am J Preventive Med**	24			**24**
**Science**	19		3	**22**

### Appendix A.5. Additional Papers 2014–2018

PubMed was searched for the terms “climate change” and “health” for the period 2014–2017 inclusive. This found 3377 papers, which were grouped by year of publication and listed alphabetically, by surname of the first author. Every 20th paper (in each year) was then examined. If a paper was found to be ineligible, successive consecutive (alphabetical) candidates were examined until at least 5% of the total maximum number for each year had been found eligible and analyzed. In total, 156 papers were scored. This sample represented 5.1% of the 3036 papers which remained after 341 of the original pool had been eliminated. More would be excluded, given a more thorough inspection. The average score of these 156 articles and their 95% confidence interval, determined by bootstrapping, was 1.29 (1.21–1.39). The average score of these papers is lower than for the papers published from 2009–2013 (1.37). Although the 95% confidence interval for the period 2014–2018 overlaps with this, there is no evidence to suggest that the more recent literature better recognizes existential risk. See Table A5 and Figure A2.

**Table A5:** This shows the number, number analyzed and scores for the 156 papers that were analyzed for the period 2014–2018, tabulated by year. Note that some of the candidate papers would be culled after further examination.

Year	Candidate Papers	Papers Analyzed	% Analyzed	Average Score
**2014**	649	34	5.2%	1.4
**2015**	639	32	5.0%	1.3
**2016**	816	43	5.3%	1.2
**2017**	813	41	5.0%	1.3
**2018**	113	6	5.1%	1.2
**Total**	**3030**	**156**	**5.1%**	**1.3**
